# *Phanerochaete chrysosporium* strain B-22, a nematophagous fungus parasitizing *Meloidogyne incognita*

**DOI:** 10.1371/journal.pone.0216688

**Published:** 2020-01-13

**Authors:** Bin Du, Yumei Xu, Hailong Dong, Yan Li, Jianming Wang

**Affiliations:** 1 College of Agriculture, Shanxi Agricultural University, Taigu, Shanxi, China; 2 Department of Horticulture, Taiyuan University, Taiyuan, Shanxi, China; Helsingin Yliopisto, FINLAND

## Abstract

The root-knot nematode *Meloidogyne incognita* has a wide host range and it is one of the most economically important crop parasites worldwide. Biological control has been a good approach for reducing *M*. *incognita* infection, for which many nematophagous fungi are reportedly applicable. However, the controlling effects of *Phanerochaete chrysosporium* strain B-22 are still unclear. In the present study we characterized the parasitism of this strain on *M*. *incognita* eggs, second-stage juveniles (J2), and adult females. The highest corrected mortality was 71.9% at 3 × 10^8^ colony forming units (CFU) mL^-1^ and the estimated median lethal concentration of the fungus was 0.96 × 10^8^ CFU mL^-1^. Two days after treatment with *Phanerochaete chrysosporium* strain B-22 eggshells were dissolved. A strong lethal effect was noted against J2, as the fungal spores developed in their body walls, germinated, and the resulting hyphae crossed the juvenile cuticle to dissolve it, thereby causing shrinkage and deformation of the juvenile body wall. The spores and hyphae also attacked adult females, causing the shrinkage and dissolution of their bodies and leakage of contents after five days. Greenhouse experiments revealed that different concentrations of the fungal spores effectively controlled *M*. *incognita*. In the roots, the highest inhibition rate for adult females, juveniles, egg mass, and gall index was 84.61%, 78.91%, 84.25%, and 79.48%, respectively. The highest juvenile inhibition rate was 89.18% in the soil. *Phanerochaete chrysosporium* strain B-22 also improved tomato plant growth, therefore being safe for tomato plants while effectively parasitizing *M*. *incognita*. This strain is thus a promising biocontrol agent against *M*. *incognita*.

## Introduction

Infections due to plant-parasitic nematodes lead to global agricultural economic losses of more than $157 billion [1] via reducing the quality and quantity of greens. Root-knot nematodes (*Meloidogyne* spp.), which comprise 98 species and parasitize almost every species of vascular plants [2], are the most economically important and destructive obligate plant-parasitic nematodes. They occur globally, especially in tropical and subtropical agricultural areas, and cause substantial yield losses (at least $77 billion yearly) to world crops [3]. *Meloidogyne incognita* is one of the most important species within genus *Meloidogyne*, and it causes severe yield losses in many cash crops (such as tomato) in greenhouse facilities, where plant-parasitic nematodes are frequently found. Although greenhouse cultivation is the main approach to produce vegetables, and an important stepping stone to modern agriculture in China, its intensive production, rich soil fertility, suitable soil temperature and moisture, and lack of effective crop rotation provide highly favorable conditions for the growth and propagation of *M*. *incognita*. After 3–5 years of cultivation under controlled conditions, crop yield loss due to *M*. *incognita* infection was 20%–40%, sometimes reaching 60% [4]. This nematode infects the roots of almost all plants cultivated in greenhouse facilities, blocks plant water and nutrient uptake due to the formation of giant cells in the roots, and facilitates infection by soil pathogenic microorganisms. Moreover, *M*. *incognita* is difficult to control because of its diverse host range, short generation time, and high reproductive rate [5]. Overall, *M*. *incognita* is known to infect 1,700 plant species [6]. In China, most greenhouse-grown vegetables are infected with *M*. *incognita*, causing annual losses of more than $400 million [7]. Therefore, *M*. *incognita* has become a prominent problem for greenhouse cropping in China.

To date, prevention and control measures against *M*. *incognita* include sanitation, crop rotation, use of organic soil amendments, trap crops, grafting, fertilization, heat-based methods, cultivation of resistant cultivars, transgenic varieties, and chemical control, among others [8, 9]. The application of chemical nematicides is the most extensively used and efficient method for the control of *M*. *incognita*. However, chemical nematicides pose serious threats to environmental and human health [10]. Thus, developing safer, more environmental-friendly, and non-toxic alternative methods for effective control of *M*. *incognita* is urgent. Biocontrol agents provide an alternative strategy for sustainable *M*. *incognita* management [11, 12]. Fungi are an important group of microorganisms that are abundant in soils, and some have been characterized for the biocontrol of plant-parasitic nematodes. However, the host ranges of nematophagous fungi are an issue for biological control, as these fungi cannot paralyze and digest all nematode species; moreover, they can be host-specific and some nematophagous fungi are good hosts and food sources for some nematode species. Nematophagous *Pleurotus ostreatus* has been confirmed to digest some nematodes, insects, and fungi via its toxin (trans-2-decenoic acid) and hypha but it can in turn be digested by *Filenchus misellus*, belonging to family Tylenchidae [13].

Nematophagous fungi reduce nematode density by parasitism, predation, or antagonism. Several species of nematophagous fungi have been isolated worldwide. These fungi include *Acremonium strictum*, *Arthrobotrys robusta*, *Catenaria auxiliaris*, *Dactylella oviparasitica*, *Hirsutella rhossiliensis*, *Nematophthora gynophila*, *Paecilomyces lilacinus*, *Pochonia chlamydosporium*, and *Trichoderma harzianum* [14–20]. Regarding their mechanisms of attack, nematophagous fungi can be categorized into four major groups: nematode-trapping, endoparasitic, egg-parasitic, and toxin-producing. Nematode-trapping fungi produce trapping devices or specialized structures, which include adhesive networks, adhesive knobs, constricting rings, and adhesive branches to capture nematodes. Endoparasitic fungi use their adhesive conidia to infect nematodes. These conidia rapidly germinate into hyphae, which can grow, digest, and penetrate the nematode body wall. Egg-parasitic fungi infect nematode eggshells by specialized pegs or appressoria. Simultaneously, these fungi usually produce extracellular hydrolytic enzymes such as proteases and chitinases that play important roles in disintegrating nematode eggshell layers. Toxin-producing fungi produce toxins that paralyze nematodes; concomitantly, they produce hyphae that can penetrate through and dissolve nematode cuticles [21].

*Phanerochaete chrysosporium*, a white-rot basidiomycete, is used for the removal of toxic pollutants from wastewater [22] and for environmental amendment. It can also decompose organic matter, such as grass seeds and pathogens from agricultural soils by composting [23], and it commonly inhabits forest litter and fallen trees. This fungus has strong degradation potential for organic compounds owing to its diverse extracellular oxidative enzymes in the growing hyphal mass [24], and it can efficiently depolymerize, degrade, and mineralize all components of plant cell walls, including cellulose, hemicellulose, and the more recalcitrant lignin [25]. Moreover, the inoculation of *P*. *chrysosporium* in greenhouse soils has been greatly effective for reducing root-knot nematode diseases and the relative disease index of root-knot nematodes on cucumber [26]. The use of *P*. *chrysosporium* in the soil can also control root wilt disease by *Fusarium oxysporum* and improve plant physiological status [27]. *Pleurotus ostreatus*, another white-rot fungi, can produce toxin droplets to attack and digest nematodes [28, 29]. The gene sequences encoding fruiting body lectins of *Pleurotus cornucopiae* are similar to that encoding the lectin of a nematode-trapping fungus [30].

The present study aimed to evaluate the efficacy of *P*. *chrysosporium* strain B-22 on the biocontrol of *M*. *incognita* under in vitro and field conditions, as this was confirmed as the main plant-parasitic nematode at the greenhouses in Taigu (Shanxi Province, China), and to assess the safety of this strain for plant growth. Our results provide basis for the development of *P*. *chrysosporium* strain B-22 as a biopesticide for the control of *M*. *incognita* in greenhouse-grown vegetables. Further, the results reported herein will allow the development of new strategies for the theoretical and practical management of *M*. *incognita*.

## Materials and methods

### Nematode inoculum preparation

Infected tomato roots were collected from a tomato plant grown in a greenhouse in the city of Taigu (Shanxi Province, China), and a single egg mass was cultured on tomato as inoculum to establish the nematode population for the experiment. The species of nematode was identified as *M*. *incognita* based on morphological and morphometrical characters [31]. Egg masses were directly extracted from infected root galls using 1% sodium hypochlorite (NaClO), and the separated eggs were gently washed with sterile water to remove the NaClO [32]. Egg masses were kept in distilled water in the dark at 25°C for 48 h. The hatched juveniles were counted under a stereomicroscope (Nikon Instruments Inc., Tokyo, Japan). The suspensions of *M*. *incognita* were diluted to approximately 200 juveniles per milliliter with distilled water. After surface sterilization, eggs, juveniles, and females were stored at 4°C for subsequent trials.

### *Phanerochaete chrysosporium* inoculum preparation

*P*. *chrysosporium* B-22 was obtained from the Plant Pathology Department at Shanxi Agriculture University in China and was cultured on potato-dextrose agar medium. *P*. *chrysosporium* was morphologically identified as in Burdsall [33]. Five days after incubation at 25°C, purified *P*. *chrysosporium* B-22 were used to produce spore suspensions for inoculation; these spore suspensions were further adjusted with autoclaved distilled water to obtain 3 × 10^8^ colony forming units (CFU) mL^-1^ for treatments, which were counted using a hemocytometer [34].

### Parasitic effect of *P*. *chrysosporium* strain B-22 on eggs, second-stage juveniles (J2), and adult females of *M*. *incognita*

Root samples infected with *M*. *incognita* were collected from greenhouse-grown tomato plants in Taigu, Shanxi Province, China. The root samples were gently washed with sterile water and further processed in the Laboratory of Nematology at Shanxi Agricultural University. *Meloidogyne incognita* egg masses and adult females were directly collected from the root samples. After surface sterilization with 1% NaCOl, adult females were suspended in sterile water and the density adjusted to 100 females mL^-1^. Egg masses were added into sterile Petri dishes with sterile water and incubated at 25°C ± 1°C for 3 days in the dark to hatch *M*. *incognita* J2. These were then surface sterilized with 1% NaCOl and incubated to obtain a 1,000 J2 mL^-1^ suspension. Egg masses were centrifuged in 1% NaOCl at 2,000 rpm for 3 min, and free eggs were collected to prepare a 3,000 eggs mL^-1^ suspension.

The parasitic effects of strain B-22 on the eggs of *M*. *incognita* were determined as described by Zhang et al. [35]. Briefly, 100 μL of *M*. *incognita* egg suspension (about 300 eggs) was placed in a sterile Petri dish containing strain B-22 spore suspension at 3 × 10^8^ CFU mL^-1^. A blank control was prepared with an equal volume of sterile water instead of the strain B-22 spore suspension. Petri dishes were incubated at 25°C ± 1°C for three days. Samples were taken for microscopic examination using a Nikon 80i microscope with an image analysis system (Nikon Instruments Inc.).

The parasitic effects of strain B-22 on *M*. *incognita* J2 were determined according to Schwartz [36]. Briefly, 100 μL of *M*. *incognita* J2 suspension (about 100 J2 individuals) was placed in a sterile Petri dish containing strain B-22 spore suspension at 3 × 10^8^ CFU mL^-1^; these Petri dishes were incubated at 25°C ± 1°C for 1 day, after which *M*. *incognita* J2 were removed and placed on a glass slide with 4% agarose for examination by fluorescence microscopy with an image analysis system (Nikon 80i microscope; Nikon Instruments Inc.). Microscopic examinations were performed 2 days later.

The parasitic effects of strain B-22 on *M*. *incognita* adult females were determined according to Dong et al. [37]. Briefly, strain B-22 was grown on water agar (WA) until the colony diameter reached near the edge of the dish. Sterile coverslips were placed in the WA, and 100 μL of adult female suspension (about 10 females) was transferred onto the coverslips and incubated at 25°C ± 1°C for five days. A control group was prepared without strain B-22. Samples were subjected to microscopic examination using the Nikon 80i microscope with an image analysis system (Nikon Instruments Inc.).

### Lethal effect of *P*. *chrysosporium* strain B-22 on *M*. *incognita* J2

Second-stage juveniles of *M*. *incognita* (about 100 J2 100 μL^-1^) were placed in sterile Petri dishes containing 1.875 × 10^7^, 3.75 × 10^7^, 7.5 × 10^7^, 1.5 × 10^8^, and 3 × 10^8^ CFU mL^-1^ of a conidial suspension of *P*. *chrysosporium* strain B-22, as determined by a Neubauer hemocytometer. The control contained sterile water and a suspension of *M*. *incognita* J2. Petri dishes were incubated at 25°C ± 1°C for 72 h, and samples were drawn at 24 h intervals to count the number of dead J2 using a Nikon stereomicroscope (Nikon Instruments Inc.).

The mortality of *M*. *incognita* J2 was determined by visual inspection of stiff individuals that stayed motionless after gently poking with a bamboo needle. The results were used to calculate the mortality and corrected mortality rates (%) of *M*. *incognita* J2. The median lethal dose (LC_50_) of strain B-22 conidia for *M*. *incognita* J2 was determined by simple linear regression analysis of the conidial concentration of strain B-22 and corrected mortality rate among J2 of *M*. *incognita*: y = ax + b, where “y” is the corrected mortality rate among J2 of *M*. *incognita* and “x” is the conidial concentration of strain B-22. Mortality (%) = (number of dead J2 in each treatment/total number of tested J2) × 100%. Corrected mortality (%) = (mortality in treatment—mortality in control)/ (1—mortality in control) *100%.

### Greenhouse evaluation of the control efficacy of *P*. *chrysosporium* strain B-22

*Phanerochaete chrysosporium* strain B-22 was incubated in potato dextrose agar plates at 25°C ± 1°C for 10 days, after which 5 mL of sterile water was pipetted onto the surface of the plates, and the spores were scraped off from the plates [38]. The spore suspension was then filtered through a fine-mesh screen (diameter 0.15 mm) to separate the spores from the hyphae, and stored at 4°C until use.

A greenhouse experiment was laid in a completely randomized design with six replications (pots) using autoclaved soil sterilized at 121°C for 2 h. The soil was individually transferred into the plastic pots (4 kg soil pot^-1^), and three seeds of the *M*. *incognita*-susceptible F1-hybrid tomato cultivar JG9002 were planted in each pot. When tomato seedlings were growing, each pot was treated with the above spore suspension at the following concentrations: 1.875 × 10^7^, 3.75 × 10^7^, 7.5 × 10^7^, 1.5 × 10^8^, and 3 × 10^8^ CFU mL^-1^. After 15 days, every pot was artificially inoculated with 1,000 *M*. *incognita* J2. All pots were maintained in the greenhouse at 25°C, under 16 h sunlight and at 65% relative humidity. Pots were fertilized weekly with approximately 3 g L^-1^ of Poly Fertisol (N:P:K = 14:10:14) and watered daily as needed. Pots inoculated with *M*. *incognita* J2—but not with the *P*. *chrysosporium* strain B-22 spore suspension—served as controls, while pots that were not inoculated with *M*. *incognita* J2 nor the *P*. *chrysosporium* strain B-22 spore suspension served as blanks.

After 10 weeks, plant and soil samples from all treatments were randomly collected and brought to the laboratory for counting nematodes in the roots and soil, and calculating root-knot index. The count of nematodes in the soil was determined as in Castillo et al. [39], and root count was determined as in Sharon et al. [40]. The extent of root galling damage was determined as previously described [41, 42]. The severity of tomato root galling was assessed on a scale from 0 to 10, where 0 = no knots on roots and 10 = all roots severely knotted or no more root system. Root-knot index = Σ (number of diseased plants in each rating * score/total number of plants investigated * highest rating) *100%. Six replications of each treatment were included.

### Safety of *P*. *chrysosporium* strain B-22 on tomato plant growth

Plant height, root length, aboveground fresh mass, and root fresh mass were measured independently in each treatment and control after 10 weeks of growth. Plant height and root length were measured with a graduated ruler, while plant fresh mass was weighed on an electronic balance.

### Statistical analyses

All experiments were repeated six times. Data were processed using Microsoft Excel 2007 (Microsoft Corp., Redmond, WA, USA) and expressed as means ± standard deviation (n = 6). The significance of differences in the counts of females, egg masses, and juveniles in the roots, tomato root gall index, and measurements of plant height, root length, aboveground fresh mass, and root fresh mass were examined using the *t*-test and one-way analysis of variance in SPSS 17.0 (SPSS Inc., Chicago, IL, USA). Statistical significance was considered at p < 0.05.

## Results

### Parasitic effect of *P*. *chrysosporium* strain B-22 on *M*. *incognita*

**Fig 1:**
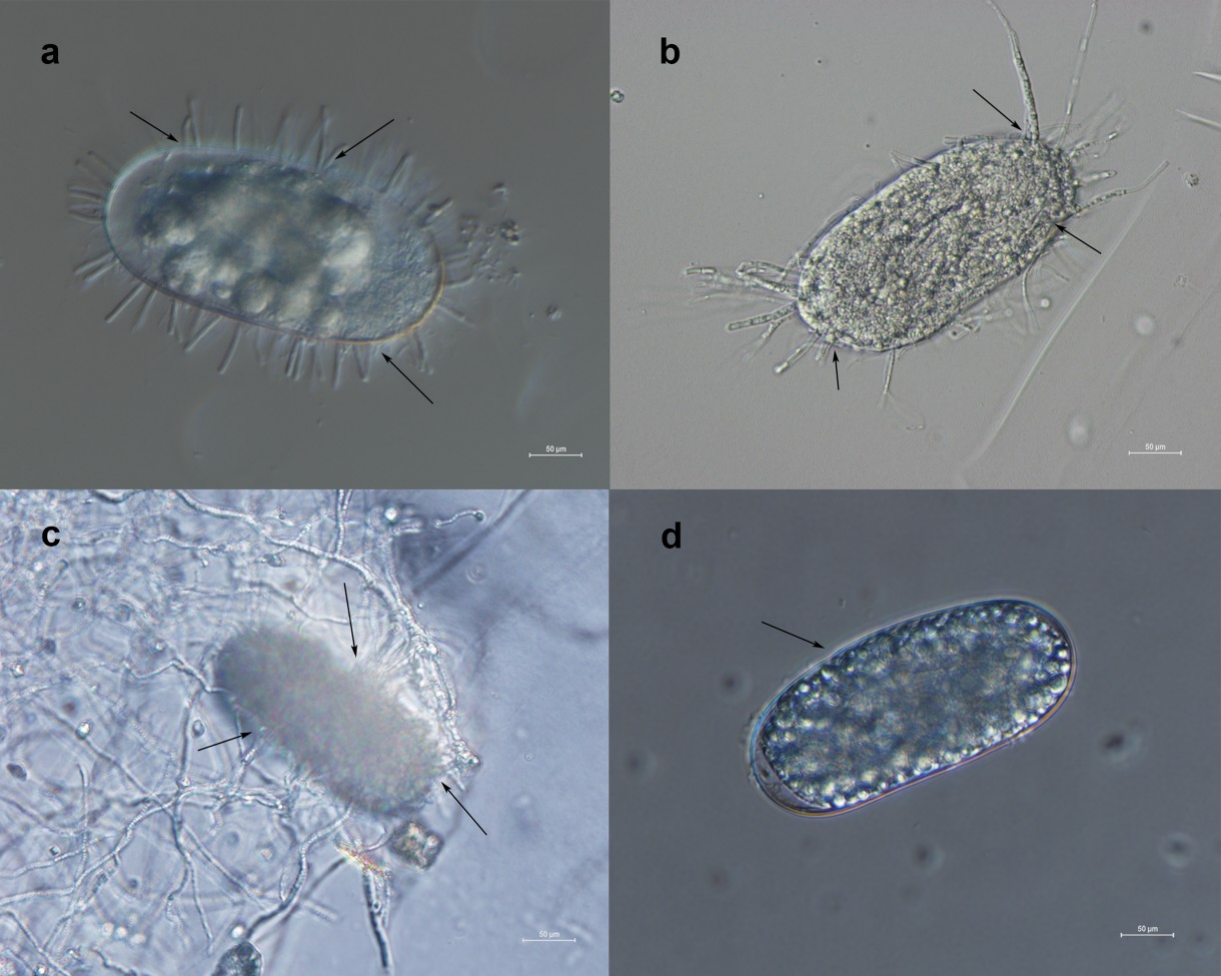
Parasitism by *Phanerochaete chrysosporium* strain B-22 on the eggs of *Meloidogyne incognita*. (a) Spores germinated, and short hyphae developed around the egg; (b) hyphae penetrated across the eggshell; (c) the eggshell was dissolved; (d) control: healthy, intact eggs. All observations were made under 40× magnification.

*Phanerochaete chrysosporium* strain B-22 parasitized the eggs of *M*. *incognita* after only two days of treatment. In the initial period of parasitism, the spores of *P*. *chrysosporium* strain B-22 were in contact and conglutinated with the eggs, and then geminated and produced short hyphae around them (Fig 1A). The fungus grew rapidly, causing the aggregation of the inner contents of the eggs, and its hyphae ran through the eggs causing shrinkage of the egg shell. More hyphae grew from the egg (Fig 1B). Over the following two days, the eggshell was deformed and shrunk further. This continued until the eggshell was completely dissolved by the fungus. The eggs were broken by the hyphae. At last, the dense mycelia of the fungus enveloped the eggs, which at the time looked abnormal and misshapen (Fig 1C). In the controls that were not inoculated with the conidia of strain B-22, the eggs of *M*. *incognita* were intact; microscopic examination confirmed that they had a smooth surface and uniform contents (Fig 1D).

**Fig 2:**
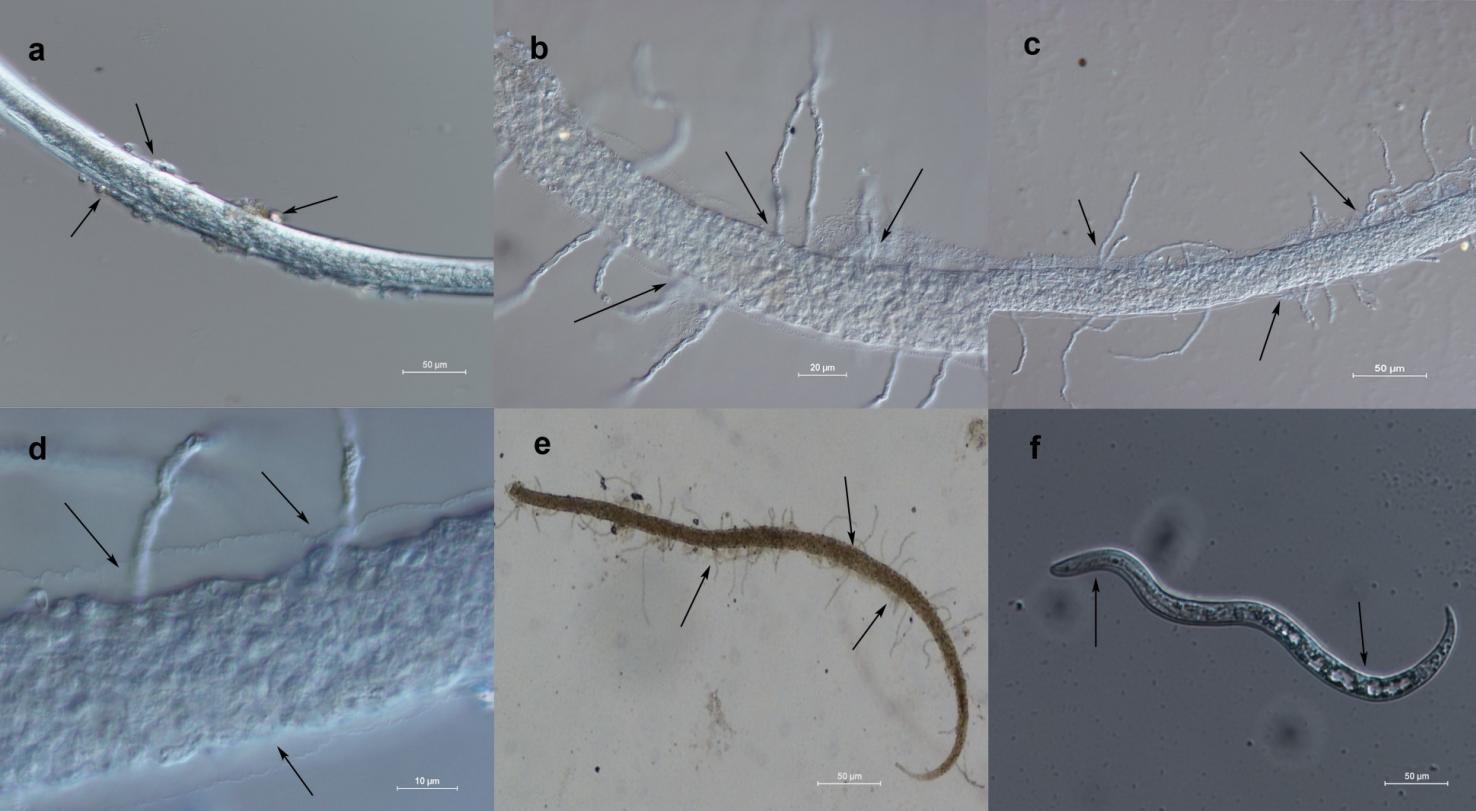
Parasitism of *Phanerochaete chrysosporium* strain B-22 on the second-stage juveniles (J2) of *Meloidogyne incognita*. (a) Spores came into contact with *M*. *incognita* J2 (20× magnification); (b) spores geminated and hyphae grew out from the bodies of *M*. *incognita* J2 (40× magnification); (c) more mycelium was produced from the bodies of *M*. *incognita* J2 (20× magnification); (d) the cuticles of *M*. *incognita* J2 were bent and shrunken (40× magnification); (e) *M*. *incognita* J2 were parasitized by *P*. *chrysosporium* strain B-22 hyphae (20× magnification). (f) Control: healthy *M*. *incognita* J2 (20× magnification).

*Phanerochaete chrysosporium* strain B-22 also parasitized *M*. *incognita* J2. After three days of incubation, *P*. *chrysosporium* strain B-22 spores were seen on the cuticle of *M*. *incognita* J2 upon microscopic examination, as observed for eggs. The conidia of the fungus developed in the body wall of nematode juveniles (Fig 2A), and the geminated spores produced hyphae from the body of the nematodes. Thus, *P*. *chrysosporium* strain B-22 grew on the surface of the cuticle of *M*. *incognita* J2 (Fig 2B). With time, *P*. *chrysosporium* strain B-22 produced more mycelium on *M*. *incognita* J2 and dissolved their cuticles, causing shrinkage and deformation of their body wall (Fig 2C). After five days of incubation, microscopic examination revealed that the cuticles of *M*. *incognita* J2 were dissolved or severely deformed. Dissolved residues of the bodies of *M*. *incognita* J2 were also clearly visible, and the cuticle was bent and shrunken (Fig 2D). Eventually, the fungus produced massive spores that developed a dense mycelium and parasitized the body surface of the nematode juveniles. The color of the cuticles was extensively altered (Fig 2E). In uninoculated controls, *M*. *incognita* J2 showed intact body wall and slow movement (Fig 2F).

**Fig 3:**
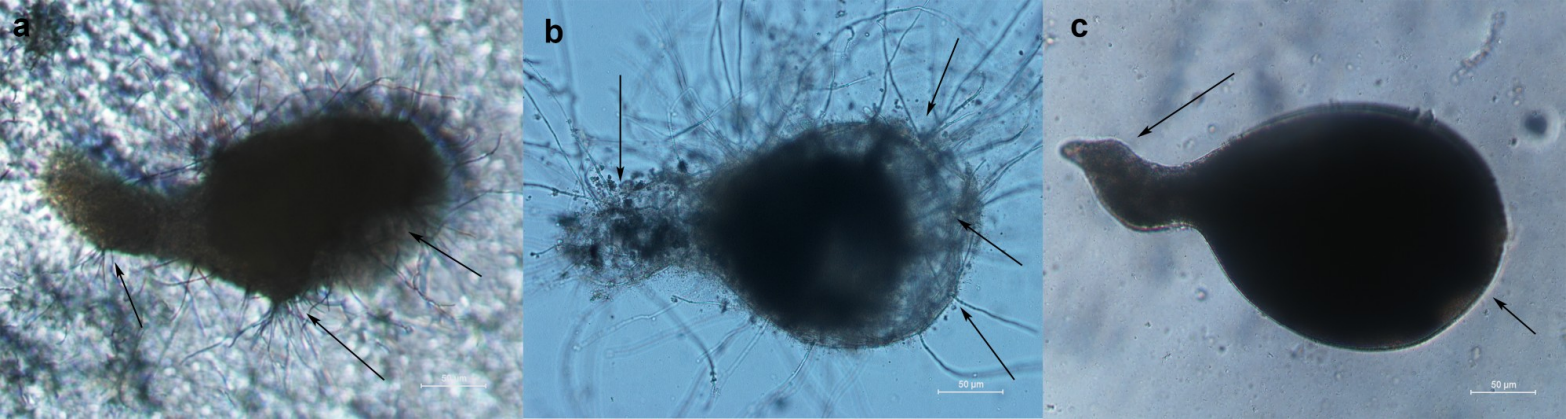
Parasitism of *Phanerochaete chrysosporium* strain B-22 on *Meloidogyne incognita* adult females. (a) Spores geminated, and the contents of *M*. *incognita* adult female bodies leaked out; (b) dense hyphae crossed *M*. *incognita* adult female bodies, which were partly dissolved. (c) Control: healthy adult female. All observations were made under 10× magnification.

*Phanerochaete chrysosporium* strain B-22 also parasitized *M*. *incognita* adult females. Upon four days of incubation, the spores of *P*. *chrysosporium* strain B-22 surrounded *M*. *incognita* adult females and stuck to their surfaces. The contents of the bodies of the females leaked out, and short hyphae grew out from their bodies (Fig 3A). After seven days of treatment, *P*. *chrysosporium* strain B-22 formed dense hyphae crossing the body walls of *M*. *incognita* adult females. Moreover, the adult female body looked severely atrophied and it was partly dissolved due to leakage (Fig 3B). In uninoculated controls, *M*. *incognita* adult females showed complete and healthy bodies with a smooth surface and obvious boundaries. Body contents of the adult females were also intact (Fig 3C).

### Lethal effect of *P*. *chrysosporium* strain B-22 on *M*. *incognita* J2

*Phanerochaete chrysosporium* strain B-22 conidial concentration and treatment time significantly influenced the mortality of *M*. *incognita* J2 (Table 1). Compared with uninoculated *M*. *incognita* J2, the mortality of *M*. *incognita* J2 infected with the fungus suspension was significantly higher (p < 0.05) in all treatments, from 1.875 × 10^7^ to 3 × 10^8^ CFU mL^-1^. In addition, mortality increased linearly with conidial concentration and time after treatment. Twenty-four hours of treatment were sufficient for causing *M*. *incognita* J2 mortality. Corrected mortality rate reached 42.8% under treatment with 3 × 10^8^ CFU mL^-1^ conidial concentration and it was significantly different (p < 0.05) from the other treatments and from the control after 24 h. Further, corrected mortality was up to 54.5% after 48 h. The highest corrected mortality (71.9%) was observed after 72 h under treatment with a conidial concentration of 3 × 10^8^ CFU mL^-1^. This mortality rate was significantly different (p < 0.05) from all other treatments.

**Table 1:** Effects of treatment time and conidial concentration of the *Phanerochaete chrysosporium* strain B-22 suspension on the mortality of second-stage juveniles (J2) of *Meloidogyne incognita*. Values within columns followed by different lowercase letters are significantly different (p < 0.05).

Time (h)	Conidial concentration (× 10^7^ CFU mL^-1^)	Mortality (%)	Corrected mortality (%)	Linear regression equation	Correlation coefficient	LC_50_ (× 10^8^ CFU mL^-1^)
24	30	50.1 ± 1.0d	42.8	Y = 0.7511 *X+ 22.91	0.8539	3.6
	15	46.7 ± 1.3f	38.9			
	7.5	40.2 ± 1.0g	31.5			
	3.75	33.2 ± 1.4h	23.4			
	1.875	31.6 ± 0.5h	21.6			
	Control	12.7 ± 1.179i	--			
48	30	62.0 ± 1.17bc	54.5	Y = 0.9246 * X + 28.19	0.9681	2.4
	15	53.6 ± 1.2de	44.4			
	7.5	47.6 ± 0.83ef	37.3			
	3.75	41.7 ± 0.65g	30.2			
	1.875	40.1 ± 0.95g	28.3			
	Control	16.4 ± 0.4i	--			
72	30	76.6 ± 1.1a	71.9	Y = 1.16 *X + 38.85	0.944	0.96
	15	65.4 ± 2.4b	58.4			
	7.5	60.2 ± 0.92c	52.2			
	3.75	50.6 ± 0.9def	40.6			
	1.875	48.9 ± 0.6f	38.6			
	Control	16.7 ± 0.35i	--			

The LC_50_ values decreased with treatment time (Table 1). Estimated LC_50_ values were 3.6, 2.4, and 0.96 × 10^8^ CFU mL^-1^ after incubation for 24, 48, and 72 h, respectively.

### Greenhouse evaluation of *P*. *chrysosporium* strain B-22 control efficacy against *M*. *incognita*

The conidial concentration of the *P*. *chrysosporium* strain B-22 suspension significantly influenced the different stages of *M*. *incognita* (Table 2). The antagonistic effect of the different conidial concentrations on the nematode was significantly greater (p < 0.05) for all treatments relative to blanks and controls. Overall, *P*. *chrysosporium* strain B-22 significantly decreased (p < 0.05) the numbers of *M*. *incognita* eggs, J2, and adult females, as well as the root gall index. The number of *M*. *incognita* J2 in the soil also decreased due to treatment with the fungal suspension. The best control efficiency on *M*. *incognita* was obtained at the conidial concentration of 3 × 10^8^ CFU mL^-1^, which was significantly different (p < 0.05) from all other treatments. The inhibition ratio of *M*. *incognita* adult females ranged between 46.15% and 84.61% in the roots, while that of the juveniles ranged from 45.57% to 78.91%, and that of the eggs from 50.39% to 84.25%. Furthermore, the inhibition ratio of the root gall index was 33.33%–79.48%; in the soil, the inhibition ratio of *M*. *incognita* J2 reached 68.51%–89.18% (Table 2).

**Table 2:** Controlling effects of *Phanerochaete chrysosporium* strain B-22 at different concentrations against *Meloidogyne incognita*. Values within columns followed by different lowercase letters are significantly different (p < 0.05.

Conidial concentration (CFU mL^-1^)	Number of females in roots (ind 2g^-1^)	Inhibition ratio (%)	Numbers of egg masses in roots (ind 2g^-1^)	Inhibition ratio (%)	Number of juveniles in roots (ind 2g^-1^)	Inhibition ratio (%)	Number of juveniles in soil (ind 2g^-1^)	Inhibition ratio (%)	Gall index in roots	Inhibition ratio (%)
3 × 10^8^	10 ± 1d	84.46	20 ± 1d	84.25	31 ± 3e	78.91	113 ± 3e	89.18	0.8 ± 0.3e	79.48
1.5 ×10^8^	19 ± 1c	70.76	39 ± 1c	69.29	49 ± 2d	66.66	144 ± 3de	86.22	1.2 ± 0.2d	69.23
7.5 × 10^7^	25 ± 2c	61.53	42 ± 2c	66.92	56 ± 2d	61.90	199 ± 7d	80.95	1.6 ± 0.3c	58.97
3.75 × 10^7^	33 ± 1bc	49.23	59 ± 2b	53.54	67 ± 2c	54.42	236 ± 7c	77.41	2.4 ± 0.3bc	38.46
1.875 × 10^7^	35 ± 2b	46.15	63 ± 3b	50.39	80 ± 4b	45.57	329 ± 9b	68.51	2.6 ± 0.2b	33.33
Control	65 ± 1a	-	127 ± 2a	-	147 ± 4a	-	1045 ± 12a	-	3.9 ± 0.7a	-
Normal	0e	-	0e	-	0f	-	0f	-	0f	-

### Safety of *P*. *chrysosporium* strain B-22 for tomato plant growth

Soil inoculation with *P*. *chrysosporium* strain B-22 was found to be safe for plant growth; in fact, this fungus significantly promoted tomato plant growth (Table 3). Plant height, root length, aboveground fresh mass, and root fresh mass were all significantly higher (p < 0.05) upon treatment with *P*. *chrysosporium* strain B-22 at different conidial concentrations, compared with blanks and controls. The highest promotion effect was achieved at the conidial concentration of 3 × 10^8^ CFU mL^-1^, which was significantly different (p < 0.05) from all other treatments. In this case, plant height was 29.5 cm, root length was 30 cm, aboveground fresh mass was 9 g, and root fresh mass was 1.5 g, while the corresponding values in the controls were 9.7 cm, 9.1 cm, 1.5 g, and 0.28 g, respectively. The highest increase rates for plant height, root length, aboveground fresh mass, and root fresh mass were 202.06%, 185.71%, 426.66%, and 292.85%, respectively (Table 3).

**Table 3:** Effects of *Phanerochaete chrysosporium* strain B-22 at different concentrations on tomato plant growth. Values within columns followed by different lowercase letters are significantly different (p < 0.05).

Conidial concentration (CFU mL^-1^)	Plant height (cm plant^-1^)	Increase (%)	Root length (cm plant^-1^)	Increase (%)	Aboveground fresh mass (g plant^-1^)	Increase (%)	Root fresh mass (g plant^-1^)	Increase (%)
3 × 10^8^	29.3 ± 2.3a	202.06	26.0 ± 4.2a	185.71	7.9 ± 1.2a	426.66	1.1 ± 0.40a	292.85
1.5 × 10^8^	24.9 ± 3.1b	156.70	20.8 ± 3.3b	128.57	4.6 ± 0.9b	206.66	0.77 ± 0.21b	175
7.5 × 10^7^	16.4 ± 2.7c	69.07	18.0 ± 5.2c	97.80	3.9 ± 0.8c	160	0.65 ± 0.33c	132.14
3.75 × 10^7^	11.9 ± 4.5d	22.68	14.1 ± 3.7d	54.94	3.3 ± 1.1d	120	0.64 ± 0.21c	128.57
1.875 × 10^7^	11.6 ± 4.8de	19.58	13.3 ± 4.1de	46.15	3.1 ± 0.5d	106.66	0.55 ± 0.34cd	96.42
Normal	11.3 ± 3.7e	16.49	12.8 ± 3.7e	40.65	3.0 ± 1.3d	100	0.50 ± 0.30d	78.57
Control	9.7 ± 2.1f	-	9.1 ± 3.4f	-	1.5 ± 0.5e	-	0.28 ± 0.27e	-

## Discussion

*Phanerochaete chrysosporium* is a white-rot fungus that has been identified as nematophagous [43]. We isolated *P*. *chrysosporium* strain B-22 as a nematophagous fungus lethal to *M*. *incognita*. Previous studies on *P*. *chrysosporium* have focused on removing toxic environmental pollutants [44]. In addition, its inoculation greatly reduced the disease index of root-knot nematode in the field and prevented wilt disease in cucumber [26] and for the control of Fusarium wilt of cut chrysanthemum [27]. Because *P*. *chrysosporium* produces many extracellular enzymes [45], it has been used in the biodegradation of lignin and nicotine in tobacco stalk [46], as well as polycyclic aromatic hydrocarbons [45] and other pollutants [47, 48]. The present study identified *P*. *chrysosporium* as a parasitic fungus of *M*. *incognita*, which was demonstrated for the first time and at different life stages of the nematode. The assay of the lethal effect by *P*. *chrysosporium* strain B-22 on *M*. *incognita* J2 demonstrated that this strain can control *M*. *incognita*. This provided further and strong evidence of the parasitism of *P*. *chrysosporium* on *M*. *incognita*.

When *M*. *incognita* eggs were treated with *P*. *chrysosporium* strain B-22, the fungus parasitized the eggs by producing hyphae, which first surrounded the eggshell and then destroyed the eggs. Some studies have shown that the egg-parasitic fungi *Verticillium suchlasporium*, *P*. *lilacinus*, *Pochonia* spp., and *T*. *harzianum* can secrete protease and chitinase, which degrade certain cyst nematode proteins to effectively destroy the nematode eggshell and later parasitize and kill the eggs [49]. As protein and chitin are the main chemical constituents of nematode cuticles and eggshells, the infection of *M*. *incognita* eggs by *P*. *chrysosporium* strain B-22 might also be involved in eggshell decomposition through the production of protease and chitinase.

*P*. *chrysosporium* strain B-22 also parasitized *M*. *incognita* J2 by producing sticky conidia that enveloped the juveniles. These were active initially but became completely destroyed after five days of incubation. Adhesive conidia of *P*. *chrysosporium* strain B-22 first stuck to the nematode body wall and produced germ tubes to invade it. Then, massive hyphae grew and crossed the nematode cuticles, causing the deformation and death of the juveniles by leakage. Moreover, the external body wall was gradually disintegrated, further indicating that *P*. *chrysosporium* strain B-22 might indeed be able to produce proteases and chitinase to degrade the nematode cuticle, as reported for the parasitic fungi *Lecanicillium psalliotae* [50]. Nematophagous *P*. *ostreatus*, another white-rot fungus, produced toxin droplets to attack nematode in 2–4 h, and its hyphae disintegrated the nematode body after 24–48 h [28, 43, 51, 52]. It is unclear whether *P*. *chrysosporium* strain B-22 employed a similar mechanism to attack *M*. *incognita* J2 juveniles prior to invasion and degradation. The decomposed relics of the external body wall of *M*. *incognita* J2 well after death indicated that this fungal strain might produce some substance(s) that kill *M*. *incognita* J2 juveniles.

When parasitizing *M*. *incognita* adult females, *P*. *chrysosporium* strain B-22 produced mycelial masses on their body surfaces. The parasitizing mycelia then crossed and digested the adult females. Our results indicated that *P*. *chrysosporium* strain B-22 parasitized *M*. *incognita* and thus effectively worked as a biocontrol agent. The mechanism of *P*. *chrysosporium* strain B-22 for controlling *M*. *incognita* involved the production of sticky conidia and hyphae to parasitize *M*. *incognita*, and on secreted extracellular enzymes and possibly other substances to digest the body of the nematode.

Overall, the present study evidenced that *P*. *chrysosporium* strain B-22 was nematophagous toward *M*. *incognita*. However, it is not clear whether other root-knot nematode species can be digested by this fungus, as the host range of nematophagous species is specific. *Pleurotus ostreatus* can trap and consume *Aphelenchus avenae* but can then be consumed by *Filenchus misellus*. *Tylencholaimus parvus* was susceptible to both *P*. *pulmonarius* and *P*. *ostreatus* whereas *A*. *avenae* was resistant to both *P*. *pulmonarius* and *P*. *ostreatus*. *Oscheius tipulae* was susceptible to *P*. *pulmonarius* but resistant to *P*. *ostreatus*. *Metarhabditis rainai* was resistant to *P*. *pulmonarius* but susceptible to *P*. *ostreatus* [53]. Thus, the nematophagous effects we found for *P*. *chrysosporium* strain B-22 may be limited to *M*. *incognita*.

Results from our greenhouse experiments clearly demonstrated that different concentrations of *P*. *chrysosporium* strain B-22 could control *M*. *incognita* in tomato. The control efficacy increased with conidial concentration of the fungal suspension. Moreover, treatment with *P*. *chrysosporium* strain B-22 significantly increased plant height, root length, and aboveground mass and root fresh mass of tomato plants. Thus, this strain effectively controlled *M*. *incognita* while improving the growth of the host tomato plants. These results indicate that *P*. *chrysosporium* strain B-22 is safe for greenhouse-grown tomato seedlings.

## Conclusions

This study characterized the parasitism of *P*. *chrysosporium* strain B-22 on the eggs, J2, and adult females of the root-knot nematode *M*. *incognita*. We also evaluated the control efficacy and safety of this fungal strain when applied to tomato plants cultured in a greenhouse. The results demonstrated the potential of *P*. *chrysosporium* strain B-22 as a promising biocontrol agent for *M*. *incognita*. However, further studies are needed to characterize the compounds responsible for the toxic effect of this parasitic fungal strain on the nematode, its physiological characteristics, and its control efficacy against other root-knot nematodes.
